# Parastomal stomach herniation complicated by gastric outlet obstruction: A case report and literature review

**DOI:** 10.1016/j.ijscr.2018.10.049

**Published:** 2018-11-11

**Authors:** Jacques Eastment, Matthew Burstow

**Affiliations:** a School of Medicine, University of Queensland, Hertson, Brisbane, QLD, 4006, Australia; b Logan Hospital, Armstrong + Meadowbrook Roads, Logan, QLD, 4131, Australia; c Griffith University, Gold Coast Campus, Southport, QLD, 4215, Australia

**Keywords:** Parastomal hernia, Gastric outlet obstruction, Stomas, surgical, Case report

## Abstract

Introduction Parastomal herniation commonly occurs following formation of an end-colostomy or ileostomy. Those patients presenting with acute complications of parastomal hernias typically undergo surgical repair. Stomach-containing parastomal hernias are rare.

Presentation of case A 92-year-old lady presented to the emergency department with vomiting and abdominal pain. She had undergone a total colectomy with end-ileostomy many years earlier. A computed tomography scan of her abdomen diagnosed gastric outlet obstruction secondary to parastomal stomach herniation. The obstruction resolved with simple nasogastric decompression and the patient did not receive surgery. She was discharged from hospital after two days.

Discussion Gastric outlet obstruction secondary to a parastomal hernia is rare. A systematic literature search found 12 previously reported cases. This is the first case managed without invasive procedures.

Conclusion For gastric outlet obstruction caused by parastomal herniation, surgeons should consider non-operative management with nasogastric decompression when the patient in question is frail and a poor surgical candidate.

## Highlights

• Gastric outlet obstruction secondary to parastomal herniation of the stomach is rare.
• It is a disease of elderly females with a history of colostomy or ileostomy formation.
• Gastric outlet obstruction should be considered in patients presenting with vomiting and a parastomal hernia.
• Frail or comorbid patients could benefit from non-operative management.

## 1 Introduction

Parastomal herniation is a frequent complication of end-stoma formation. It occurs most commonly following fashioning of an end-colostomy [1]. Increased rates of herniation are observed in patient groups with advanced age, obesity and peristomal complications in the post-operative setting [2,3]. Surgical management is typically reserved for patients experiencing acute complications of their hernia. A meta-analysis comparing surgical repair techniques for parastomal herniation concluded that herniorraphy with mesh was associated with significantly lower recurrence risk compared to primary repair without mesh [4].

We present a case report of a woman who presented to a large metropolitan hospital emergency department with gastric outlet obstruction secondary to a stomach-containing parastomal hernia. To our knowledge, this is the first case report of such a presentation not managed by way of surgery or procedural intervention. This case has been reported as per the Surgical case report guidelines (SCARE) criteria [5].

## 2 Presentation of case

A frail 92-year-old Caucasian lady was referred to the emergency department from her nursing home with high volume vomiting. She had been unwell for three days with associated nausea, anorexia and generalised abdominal pain. The vomitus was described as black and watery without bloodstaining. The patient’s abdomen had become distended over the last 24 h.

The patient had a significant surgical history. She had undergone an emergency laparotomy and Hartmann’s procedure 17 years earlier for an obstructing sigmoid adenocarcinoma. The patient then had a total colectomy and fashioning of an end ileostomy. She had also received a laparoscopic cholecystectomy. Her medical history included a cerebrovascular accident, osteoarthritis and reflux disease. She had never smoked.

**Fig. 1:**
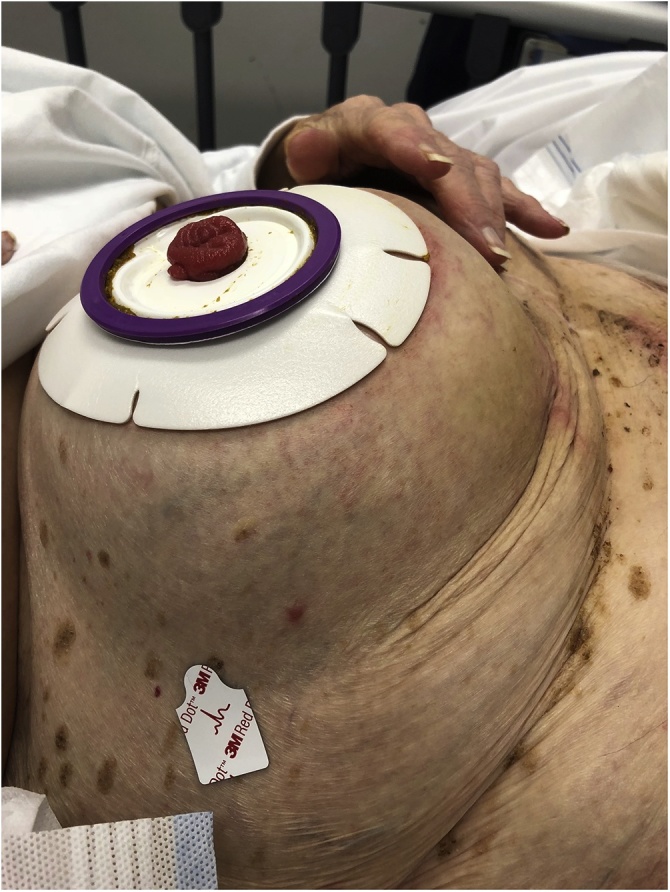
Photograph of the patient’s abdomen demonstrating a large parastomal hernia and healthy ileostomy muscosa.

On examination the patient was a thin woman who looked unwell and dehydrated. She was haemodynamically stable and afebrile in the emergency department. Her abdomen was grossly distended, particularly around her ileostomy. There was a tense, circumferential swelling around the stoma, which was functional with a healthy mucosa (Fig. 1). A tender parastomal hernia was evident from abdominal palpation. There was no peritonism. Examination of the hernia seemed to cause the patient to vomit.

**Fig. 2:**
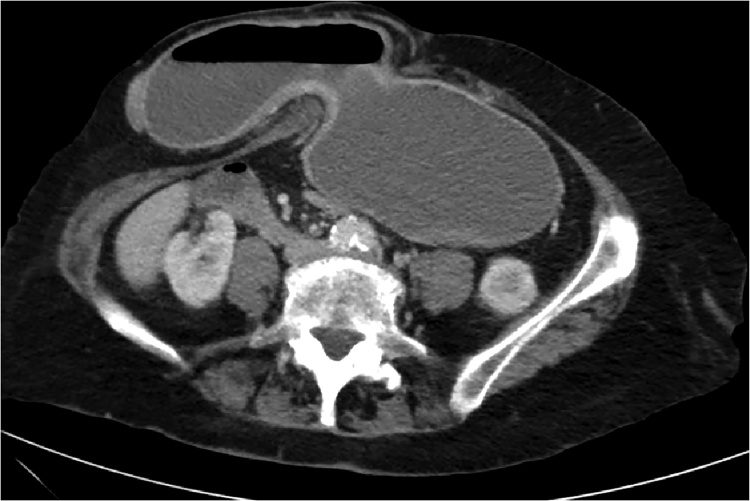
A transverse plane view of the patient’s abdomino-pelvic commuted tomography scan. The parastomal hernia sac is to the right of midline and contains the distal stomach. The parastomal hernia defect measured 43 mm.

**Fig. 3:**
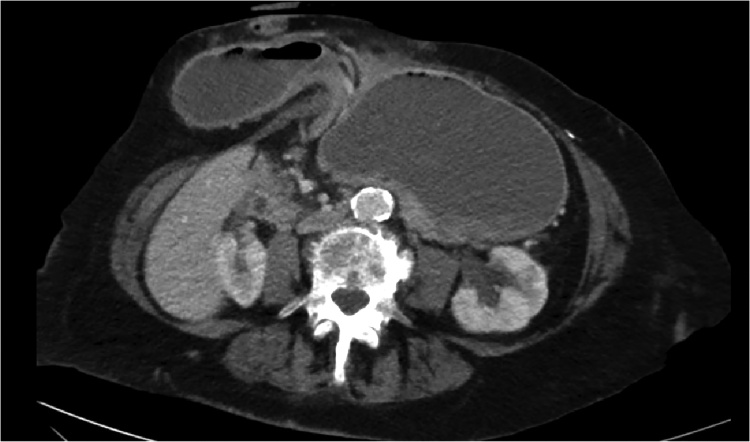
A transverse plan view of the patient’s commuted tomorgraphy abdominal scan. The pylorus is collapsed and can be appreciated returning to the peritoneal cavity. This image is consistent with gastric outlet obstruction.

**Fig. 4:**
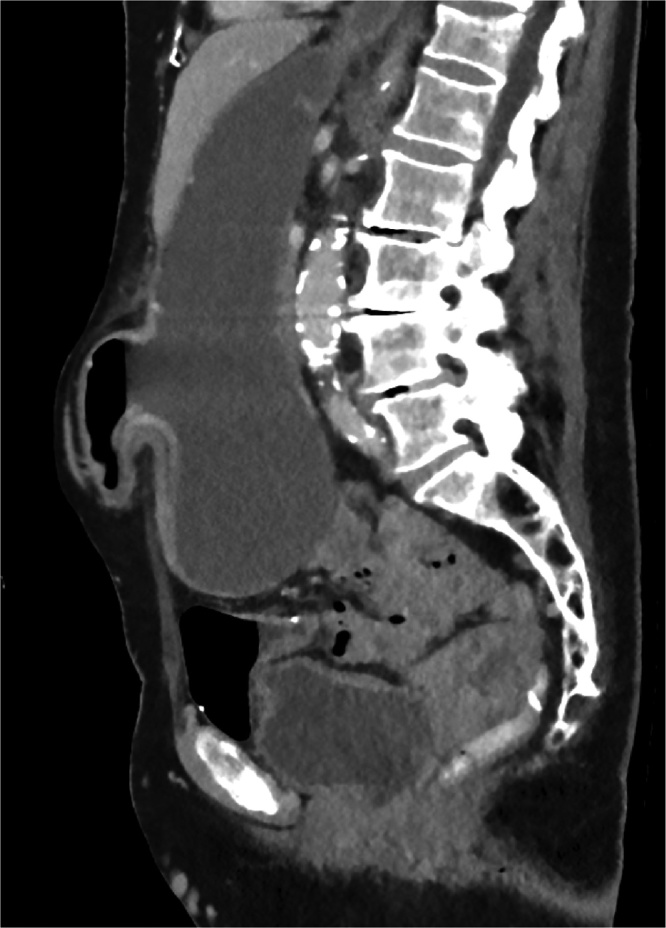
Sagittal plane slice from the patient’s commuted tomography abdominal scan. The stomach is distended and enters the parastomal hernia sac with air fluid levels present.

Basic haematological tests were completed. The patient had normal electrolytes but had developed an acute kidney injury with a serum creatinine of 130 mmol/L. A prompt abdomino-pelvic computed tomography (CT) scan was organised. It demonstrated a parastomal hernia containing the distal half of the gastric body and a collapsed pylorus (Fig. 2, Fig. 3, Fig. 4). The lower oeseophagus was distended and fluid filled in keeping with gastric outlet obstruction. The hernial sac did not contain any bowel loops. The parastomal hernia measured 43 mm in diameter.

Nasogastric decompression and intravenous fluid resuscitation was commenced in the emergency department. The nasogastric tube drained one litre of fluid within one hour of insertion. The patient felt much improved after this. Her pain settled and her abdominal distension was less apparent. The parastomal hernia was smaller and no longer tense or tender to palpate. The patient and her family were reluctant about a further abdominal surgery to repair her hernia. Given the patient’s age, comorbidities and clinical improvement with simple measures, a decision was taken to manage this presentation non-operatively.

The patient was admitted under the care of an acute surgical unit. Over the following 48 h, she improved clinically and biochemically. The patient’s pre-renal acute kidney injury responded to intravenous resuscitation and careful fluid balancing. Her nasogastric tube was removed after one day in hospital and she tolerated a diet without recurrent vomiting. Her parastomal hernia remained palpable but there was no evidence of further gastric outlet obstruction. The patient was then discharged from hospital without any complications. She has not required re-hospitalisation at one-month follow-up.

## 3 Discussion

Parastomal herniation of the stomach is rare. A systematic literature search of EMBASE and MEDLINE databases was conducted using the terms ‘parastomal hernia’ and ‘stomach’. There were 37 published manuscripts found in this search. Following perusal of each abstract and removal of duplicates, nine articles were identified for initial inclusion. Bibliographic analysis helped authors identify two further manuscripts. In total, 12 other case reports of gastric parastomal herniation were identified from 11 previous publications (Table 1) [[6], [7], [8], [9], [10], [11], [12], [13], [14], [15], [16]].

**Table 1:** Characteristics of case reports of gastric parastomal hernia repair.

First Author (reference)	Year of publication	Age (yrs)	Sex	Previous surgery	Time since surgery	Management	Operative findings	Negative outcome(s)
Figiel (6)	1967	76	F	Transverse loop colostomy	5 days	Laparotomy + herniorraphy (no mesh)	Viable stomach, ischaemic bowel	Death
McAllister (7)	1991	91	F	Hartmann’s procedure (end colostomy)	3 years	Laparotomy + stomal transposition + herniorraphy (no mesh)	Viable stomach	None
Ellingson (8)	1993	77	F	Hartmann’s procedure (end colostomy)	23 years	Laparotomy + herniorraphy (no mesh)	Stomach was already reduced	None
Bota (9)	2012	41	F	Panproctocolectomy (end ileostomy)	10 years	Laparotomy + herniorraphy (mesh)	Viable stomach and small bowel	Mesh infection
Ilyas (10)	2012	93	F	Hartmann’s procedure (end colostomy)	4 years	Laparotomy + herniorraphy (no mesh)	Viable stomach	None
Ramia-Angel (11)	2012	64	F	Abdominoperineal resection (end colostomy)	17 years	Gastric decompression + gastroscopy	Ischaemic fundal changes	None
Marsh (12)	2012	81	M	Rectal resection (end colostomy)	19 years	Laparotomy + stomach repair + stomal transposition + enlargement of hernia defect	Perforated stomach	Wound infection
Barber-Millet (13)	2014	69	F	Hartmann’s procedure (end colostomy)	9 years	Laparotomy + stomal reposition with mesh + herniorraphy	Viable stomach	None
Bull (14)	2017	85	F	Loop colostomy	10 years	Laparotomy + colostomy excision + herniorraphy + ileostomy	Stomach was already reduced	None
Garza (15)	2017	81	M	Hartmann’s procedure (end colostomy)	6 years	Laparoscopic herniorraphy (mesh)	Viable stomach and small bowel	None
Vierstraete (16)	2018	74	F	Colostomy refashioning	1 year	Laparotomy + stomal transposition + herniorraphy	Viable stomach	Gastroparesis
Vierstraete (16)	2018	69	F	Pelvic exenteration (end colostomy)	5 years	Laparotomy + herniorraphy (mesh)	Viable stomach and small bowel	None
Eastment (current study)	2018	91	F	Total colectomy (end ileostomy)	16 years	Non operative gastric decompression	N/A	None

Bracketed numbers correspond to references in bibliography. F = female, M = male.

Although there are only a handful of case reports on which to draw conclusions, this appears to be a disease of the elderly with the average age of recorded patients being 76 years. The majority of cases occurred in patients with colostomies. It has been postulated that this group is at risk of parastomal gastric herniation because of their acquired fascial defects and lax gastric ligaments [8]. Excluding one patient who developed a parastomal hernia five days after transverse colostomy formation, the average time from stoma formation surgery to gastric herniation was ten years.

The current study is the only case in the literature in which gastric outlet obstruction secondary to a stomach-containing parastomal hernia was managed without invasive intervention. One previous patient had undergone nasogastric decompression and endoscopic gastroscopy to assess the viability of the stomach [11]. These are the only cases managed non-operatively. The majority of patients were treated surgically with or without mesh repair and stoma transposition (Table 1). Despite the elderly age group, most of these surgical patients had positive outcomes. There was one death following laparotomy in 1967(6). However, this patient was found to have widespread mesenteric ischaemia and unviable small and large bowel at operation. Modern case reports present better outcomes with two post-operative infections and no mortality [9,12].

Most historical cases document viable stomach tissue at the time of operation. There are two cases of radiological gastric pneumatosis but both patients avoided stomach resection [10,16]. One patient had a partly ischaemic stomach secondary to mesenteric thrombosis but survived conservative management for this [11]. In two patients, the stomach was reduced from the parastomal hernia at time of surgery – suggesting that a period of non-operative management in the first instance may be advisable [8,14].

To our knowledge, the current study is the first case of gastric outlet obstruction secondary to a parastomal hernia that was managed without any invasive procedures. This approach is simple and low risk. Whilst our patient is at risk of recurrence, she has been spared the morbidity of another abdominal operation and remains clinically well.

## 4 Conclusions

Parastomal herniation of the stomach is a rare but important cause of gastric outlet obstruction. It is a disease of elderly females with a history of colostomy or ileostomy formation. Although most patients seem to have positive operative outcomes, surgeons should consider non-operative management with nasogastric decompression when the patient in question is frail and a poor surgical candidate.

## Conflicts of interest

No conflicts of interest to declare.

## Sources of funding

No sources of funding for this research.

## Ethical approval

Single patient observational case report with express written permission from the patient involved. This satisfies ethical requirements as per local institutional guidelines.

## Consent

Written consent from the patient has been received and this communication can be readily produced to the Editor in Chief if required. There are no conditions or caveats to this consent.

## Author contribution

Jacques Eastment: conceptualisation, data curation, formal analysis, investigation, methodology, project administration, software, validation, visualisation, writing – original and subsequent drafts.

Matthew Burstow: conceptualisation, methodology, supervision, writing – review and editing.

Not applicable: funding acquisition.

## Registration of research studies

UTN: U1111-1222-0063.

Registry: Australian New Zealand Clinical Trials Registry (ANZCTR).

## Guarantor

Dr Jacques Eastment MBBS (Hons) BSc.

## Provenance and peer review

Not commissioned, externally peer reviewed.

## References

[1] Carne P.W.G., Robertson G.M., Frizelle F.A. (2003). Parastomal hernia. BJS.

[2] Ripoche J., Basurko C., Fabbro-Perray P., Prudhomme M. (2011). Parastomal hernia. A study of the French federation of ostomy patients. J. Visc. Surg..

[3] De Raet J., Delvaux G., Haentjens P., Van Nieuwenhove Y. (2008). Waist circumference is an independent risk factor for the development of parastomal hernia after permanent colostomy. Dis. Colon Rectum.

[4] Hansson B.M.E., Slater N.J., van der Velden A.S., Groenewoud H.M.M., Buyne O.R., de Hingh I.H.J.T. (2012). Surgical techniques for parastomal hernia repair: a systematic review of the literature. Ann Surg..

[5] Agha R.A., Fowler A.J., Saeta A., Barai I., Rajmohan S., Orgill D.P. (2016). The SCARE statement: consensus-based surgical case report guidelines. Int. J. Surg..

[6] Figiel L.S., Figiel S.J. (1967). Gastric herniation as a complication of transverse colostomy. Radiology.

[7] McAllister J.D.M., D’Altorio R.A.M. (1991). A rare cause of parastomal hernia: stomach herniation. South. Med. J..

[8] Ellingson T.L., Maki J.H., Kozarek R.A., Patterson D.J. (1993). An incarcerated peristomal gastric hernia causing gastric outlet obstruction. J. Clin. Gastroenterol..

[9] Bota E., Shaikh I., Fernandes R., Doughan S. (2012). Stomach in a parastomal hernia: uncommon presentation. BMJ Case Rep..

[10] Ilyas C., Young A., Lewis M., Suppia A., Gerotfeke R., Perry E. (2012). Parastomal hernia causing gastric emphysema. Ann. R. Coll. Surg. Engl..

[11] Ramia-Ángel J., De la Plaza R., Quiñones-Sampedro J., Veguillas P., García-Parreño J. (2012). Gastrointestinal: gastric incarceration in parastomal hernia. J. Gastroenterol. Hepatol..

[12] Marsh A.K., Hoejgaard M. (2013). Incarcerated and perforated stomach found in parastomal hernia: a case of a stomach in a parastomal hernia and subsequent strangulation-induced necrosis and perforation. J. Surg. Case Rep..

[13] Barber-Millet S., Pous S., Navarro V., Iserte J., García-Granero E. (2014). Parastomal hernia containing stomach. Int. Surg..

[14] Bull N., Chan D.L., Ravindran P., Sano S.D., White S.I. (2017). Gastric outlet obstruction secondary to parastomal hernia: case report and literature review. Aust. N. Z. J. Surg..

[15] Garza A.C.N.M., Jaurrieta R., Rangel Rios H., Salgado C., Chapa L. (2017). Laparoscopic two-mesh repair of a giant parastomal hernia. Surg. Endosc..

[16] Vierstraete M., Van de Putte D., Pattyn P. (2018). Symptomatic gastric involvement in a parastomal hernia: uncommon presentation. Acta Chir. Belg..

